# Trait Mindfulness Across Adulthood: Can Mindfulness Buffer Physiological Reactivity to Spousal Distress?

**DOI:** 10.1093/geroni/igaf122.4032

**Published:** 2025-12-31

**Authors:** Skylar Weiskittel, Stephanie Wilson

**Affiliations:** The University of Alabama at Birmingham, Birmingham, Alabama, United States; The University of Alabama at Birmingham, Birmingham, Alabama, United States

## Abstract

Older adults tend to have higher trait mindfulness—the capacity to sustain attention to present experiences without judgement—compared to younger adults. This may reflect age-related improvements in emotion regulation due to life experience and a shift in perceived time horizons. Higher trait mindfulness has been linked to reduced physiological reactivity to psychosocial stressors, promoting both physical and mental well-being. However, it remains unclear whether trait mindfulness protects older adults from blood pressure reactivity during exposure to a spouse’s emotional distress, an important but understudied marital stressor. To examine this, 204 community adults ages 25–90 (N = 102 couples) reported their trait mindfulness and had their blood pressure measured before and after an emotional disclosure task. First, age was positively correlated with trait mindfulness (r = 0.40, p < 0.001), replicating prior work. Controlling for the fixed effects of sex, BMI, tobacco, comorbidities, and education, multilevel models indicated that trait mindfulness did not alter blood pressure reactivity (ps > .361). Older adults also did not differentially benefit from mindfulness in terms of systolic or diastolic blood pressure reactivity (ps > .230). However, older adults did show diminished diastolic reactivity compared to younger adults (B = -0.088, SE = 0.029, p=.003), apart from their mindfulness levels. Thus, although older adults reported greater mindfulness and experienced dampened diastolic reactivity to their partner’s emotional distress, mindfulness did not account for or amplify this physiological advantage. Future research should examine distinct facets of trait mindfulness and explore other psychosocial factors that may confer benefits to older adults under stress.

